# Development of INDEL Markers for Genetic Mapping Based on Whole Genome Resequencing in Soybean

**DOI:** 10.1534/g3.115.022780

**Published:** 2015-10-23

**Authors:** Xiaofeng Song, Haichao Wei, Wen Cheng, Suxin Yang, Yanxiu Zhao, Xuan Li, Da Luo, Hui Zhang, Xianzhong Feng

**Affiliations:** * Key Laboratory of Systems Biology in Universities of Shandong, College of Life Science, Shandong Normal University, 250014 Jinan, China; † Key Laboratory of Soybean Molecular Design Breeding, Northeast Institute of Geography and Agroecology, Chinese Academy of Sciences, 130102 Changchun, China; ‡ Key Laboratory of Synthetic Biology, Institute of Plant Physiology and Ecology, Shanghai Institutes for Biological Sciences, Chinese Academy of Sciences, 200032, China; § School of Life Sciences, Sun Yat-Sen University, 510275 Guangzhou, China

**Keywords:** soybean, whole genome resequencing, INDEL markers, *crinkly leaf* mutant, genetic mapping

## Abstract

Soybean [*Glycine max* (L.) Merrill] is an important crop worldwide. In this study, a Chinese local soybean cultivar, Hedou 12, was resequenced by next generation sequencing technology to develop INsertion/DELetion (INDEL) markers for genetic mapping. 49,276 INDEL polymorphisms and 242,059 single nucleotide polymorphisms were detected between Hedou 12 and the Williams 82 reference sequence. Of these, 243 candidate INDEL markers ranging from 5–50 bp in length were chosen for validation, and 165 (68%) of them revealed polymorphisms between Hedou 12 and Williams 82. The validated INDEL markers were also tested in 12 other soybean cultivars. The number of polymorphisms in the pairwise comparisons of 14 soybean cultivars varied from 27 to 165. To test the utility of these INDEL markers, they were used to perform genetic mapping of a *crinkly leaf* mutant, and the *CRINKLY LEAF* locus was successfully mapped to a 360 kb region on chromosome 7. This research shows that high-throughput sequencing technologies can facilitate the development of genome-wide molecular markers for genetic mapping in soybean.

Cultivated soybean [*Glycine max* (L.) Merrill] is an economically important crop that provides a large amount of the world’s plant protein and oil. Most of the important agronomic traits of soybean are usually quantitative including yield, quality, and resistance to environmental stresses (Bolon *et al.* 2010; Lee *et al.* 2004; Liang *et al.* 2010; Xing *et al.* 2012; Zhang *et al.* 2004). Soybean has more than 46,430 protein-coding genes, nearly 75% of which exist as multiple homologs (Schmutz *et al.* 2010). The complexity of the soybean genome makes it difficult to perform studies on gene functions. It is crucial that adequate molecular markers for genotypes of interest be developed that can be used for mapping and marker-assisted selection. However, most of the available soybean molecular markers are based on polymorphisms among popular studied cultivars. Screening for polymorphisms between new local cultivars and well studied ones will expand the scope of the molecular markers available and facilitate soybean genetic studies.

Whole genome sequencing projects bring significant changes to traditional genetic mapping. Next generation sequencing (NGS) technologies have unlocked a new era of plant and animal genome sequencing, providing cost-effective sequence data for developing genetic markers that can be used in plant breeding and genetic studies. Single nucleotide polymorphisms (SNPs) are the most abundant markers revealed by NGS technologies, and have been widely used for genetic studies because of their relative technical simplicity (Ganal *et al.* 2009; Kumar *et al.* 2012; Rafalski 2002; Zhou *et al.* 2014). INsertion/DELetion (INDEL) polymorphisms are the second most abundant form of genetic variations after SNPs in human and plants (Pena and Pena 2012). They are ubiquitous in genomes, occurring nearly as frequently as SNPs, but with great diversity in size (Montgomery *et al.* 2013; Mullaney *et al.* 2010; Pacurar *et al.* 2012). INDELs can be distinguished easily based on their size. INDELs have previously been used as powerful taxon diagnostics and phylogenetic markers, and are becoming popular as genetic markers for mapping and other genetic studies in crops (Li *et al.* 2015; Lister *et al.* 2009; Liu *et al.* 2012; Moghaddam *et al.* 2014; Pacurar *et al.* 2012; Schneeberger and Weigel 2011; Wu *et al.* 2014; Yamaki *et al.* 2013). With the release of soybean genome information, soybean genomes are being sequenced at an unprecedented rate; hundreds of wild and cultivated soybean genomes have now been published (Kim *et al.* 2010; Lam *et al.* 2010; Zhou *et al.* 2015). INDELs can be easily detected with the massive amount of genomic information that is available.

A large number of soybean molecular markers, including restriction fragment length polymorphisms (RFLPs), amplified fragment length polymorphisms (AFLP), simple sequence repeats (SSRs) and SNPs, have been collected in SoyBase (http://SoyBase.org). Hwang *et al.* (2009) examined the allelic diversity of 1238 SSR markers among 23 soybean cultivars or lines and a wild accession. Song *et al.* (2010) created the BARCSOYSSR database containing 33,065 SSRs with a high likelihood of containing polymorphisms, which has been a useful tool for soybean breeders and geneticists as a source of DNA markers. Hyten *et al.* (2010) added 2500 additional SNP markers to the integrated map and created a universal linkage panel for quantitative trait locus (QTL) mapping with 1536 SNPs through deep resequencing of a reduced representational library. Song *et al.* (2013) developed the SoySNP50K array by resequencing six cultivated and two wild soybean genotypes. Recently, Lee *et al.* (2015) identified more than four million high-quality SNPs from the genome resequencing of 48 soybean accessions and created the Axiom SoyaSNP array containing 180,961 SNPs. Compared with the great progress in SNP marker development in soybean, there are few INDEL records in the public database, and it is essential to fill this vacancy using information obtained from NGS data.

Hedou 12 is a widely planted soybean cultivar in Shandong province, China. With its high and stable yield, it has been classified as a reference cultivar for Chinese soybean variety certification for decades. Its yield is over 4000 kg/hectare (ha) in the field, and it has a high yield record of 4531.5 kg/ha, making it a representative high yield cultivar in China. It is also resistant to soybean mosaic virus, *Phytophthora megasperma*, and *Peronospora manschurica*. A gamma ray mutagenesis population of Hedou 12, with more than 60,000 independent lines, has been generated from 2008–2014 in our lab, from which 2742 visible mutants have been screened. In order to identify the mutated genes of these mutants, a reliable anchor markers system is needed for future genetic mapping studies. In this study, we resequenced Hedou 12, identified a large number of INDELs and SNPs with whole genome resequencing data, and developed a small number of INDELs as anchor markers for preliminary mapping. With these markers, a tested mutated locus of the *crinkly leaf* mutant was successfully mapped to a small genomic region of chromosome 7.

## Materials and Methods

### Plant materials

Fourteen soybean cultivars were used for confirmation of INDEL polymorphism, including nine Chinese cultivars coming from seven provinces of China, and five American cultivars. The nine Chinese cultivars were Hedou 12, Jilin 35, Wandou 28, Jindou 21, Fendou 33, Shidou 111, Andou 1311, Zhonghuang 30, and Zhoudou 30. The five American cultivars were Williams 82, Jack, Essex, Forrest, and HVE. The *crinkly leaf* mutant was screened from our Hedou 12 gamma ray irradiated mutagenesis population. The mutant was backcrossed to wild-type Hedou 12 for four generations to purify the genetic background before it was subjected to further analyses. One homozygous mutant plant was crossed to Williams 82, and the progeny of F1 plants were grown in the field at Shandong Normal University, Jinan, China. The mutants that segregated from the F2 population were collected for genotyping. The fine mapping of *crinkly leaf* was conducted using the scheme proposed by Jander *et al.* (2002).

### Resequencing Hedou 12 on an Illumina Hisequation 2000 platform

The genomic DNA of Hedou 12 was extracted from one single plant’s leaf tissue and sonicated using a Biorupter (Diagenode) to generate DNA fragments of ∼200 to 600 bp. The resulting fragments were blunt ended, ligated with adaptors, and selected on a 1.0% agarose gel. The 350–600 bp fractions were purified and resequenced using the Illumina Hisequation 2000 platform (Illumina Inc, San Diego, CA). Reads of 100 nt in length were generated from paired-end libraries of ∼400 ± 100 bp insert sizes.

### Sequence alignment, and INDEL and SNP calling

Low quality reads were deleted or trimmed by a base-quality Q score in Phred scale < 20 and read size <60 bp from raw data using Perl scripts. The cleaned data were mapped to the reference genome of Williams 82 retrieved from Phytozome (http://www.phytozome.org/; Williams 82 V1.01) using Burrows-Wheeler Aligner (BWA) software (version 0.5.8c, Li and Durbin 2010) with no more than three mismatches. INDELs and SNPs were called using SAMtools (version 0.1.12a) (Li *et al.* 2009; Wang *et al.* 2011). Only INDELs that met all the following criteria were retained: a) read depth ≥ 5; b) minimum mapping quality ≥ 20; and c) the length of INDELs ≥ 5 bp. For SNPs, the criteria were: a) read depth ≥ 5; b) minimum mapping quality ≥ 20; c) the reference or variant allele was not an N; and d) no more than one variant allele existed.

### Selection of INDEL markers

We chose 195 detected INDELs that were distributed across all 20 chromosomes as candidate anchor markers for preliminary mapping. In order to test the INDEL markers’ application potential for fine-scale mapping and marker screening efficiency, we chose 48 candidate INDEL loci in three regions for fine-scale INDEL marker screening. This encompassed 37.3 Mb in three chromosomes: Gm01 (from MOL0849 to MOL1147, 31 candidate loci in 34.2 Mb), Gm02 (from MOL1223 to MOL1193, 11 candidate loci in 1.3 Mb), and Gm11 (from MOL1183 to MOL1175, six candidate loci in 1.7 Mb). To be readable in polyacrylamide gels after electrophoresis, the selected candidate INDEL polymorphisms were 5–50 bp in length. The upstream and downstream sequences of the INDEL loci were collected from Phytozome for polymerase chain reaction (PCR) primer design. The primer size was set from 18–30 bp and the GC content was set from 40–60%. The maximum Tm difference between forward and reverse primers was set to 3°. The size of PCR products ranged from 100–600 bp depending on primer design and polymorphism readability after polyacrylamide gel electrophoresis.

### DNA extraction and INDEL marker analysis

Genomic DNA was extracted using the DNeasy Plant Mini Kit (Qiagen). Template genomic DNA of ∼100 ng was amplified in a 10 μL volume reaction containing 0.5 U Taq polymerase (TAKARA, rTaq), 1×PCR Buffer (with 2.0 mM Mg^2+^), 100 μM dNTP each, and 0.5 μM of each primer. PCR conditions were as follows: 94° for 3 min followed by 35 cycles of 94° for 30 sec, 54 -58° for 30 sec, and 72° for 30 sec, with a final extension of 5 min at 72°. The PCR products were separated on a 12% native-polyacrylamide gel by electrophoresis. The gels were stained with ethidium bromide for 5 min, and visualized and photographed on an ultraviolet transmission analyzer.

### Inverse PCR

2 μg of genomic DNA was digested in a 25 μL volume reaction containing 20 units *Eco*R I (NEB) and 1× NEB buffer 4 for 3 hr, incubating at 65° for 20 min. The digested genomic DNA was ligated in a 200 μL volume reaction containing 80 units T4 ligase (NEB) and 1× ligase buffer at 22° overnight. Next, 20 μL 3M NaAc and 500 μL ethyl alcohol were added to the ligation products, centrifuged at 12,000 rpm for 8 min, the precipitate was dried, and 50 μL double distilled water was used to dissolve the DNA. The PCR reaction and conditions were the same as described in above but with 2 min extension. The inverse PCR primers were OL2578 (5’-GGACTACCAGATCTCTAGCAGGC-3’) and OL2579 (5’-ATGAAGAAGTTGCTCGTCTATTGTG-3’). Primers for the validation of the inverse PCR result were OL2623 (5’-AACATATTCCTGGCTGACATCAG-3’) and OL2624 (5’-CATATTTAACTGGTCGGTGAAGC-‘3).

### Cluster analysis

A cluster analysis of the 14 cultivars was performed by MEGA 5.2.1 (Kumar and Nei 2007).

### Data availability

The polymorphism information of INDELs and SNPs between Hedou 12 and Williams 82 was deposited in SoyBase (http://soybase.org/projects/SoyBase.B2014.01.php).

## Results and Discussion

### Identification of INDELs between Hedou 12 and Williams 82

From sequencing the genome of Hedou 12, 420,767,262 reads were generated, corresponding to about 42 gigabases (Gb) of raw sequence data. After quality control, 333.6 million valid reads were recorded, corresponding to 32.3 Gb of sequence data. Using the BWA software with default parameters, 27.1 Gb of sequence (accounting for 83.8% of the valid data) was aligned to the genome of Williams 82, covering 91.7% of the reference sequence at a 30.3× sequencing depth. 49,276 candidate INDELs and 242,059 SNPs were called between the Hedou 12 genome and the Williams 82 reference sequence using the SAMtools software. Of the 49,276 detected INDELs, 43 were heterozygous loci in Hedou 12 and the remaining 49,233 were recognized as homozygous loci (29,586 deletions and 19,647 insertions). 620 detected INDELs were located on unanchored sequence scaffolds. Finally, 29,141 deletions and 19,472 insertions were retained. These deletions and insertions with 242,059 SNPs between Hedou 12 and reference sequence Williams 82 will also be useful tools for genetic mapping between these two cultivars.

**Figure 1:**
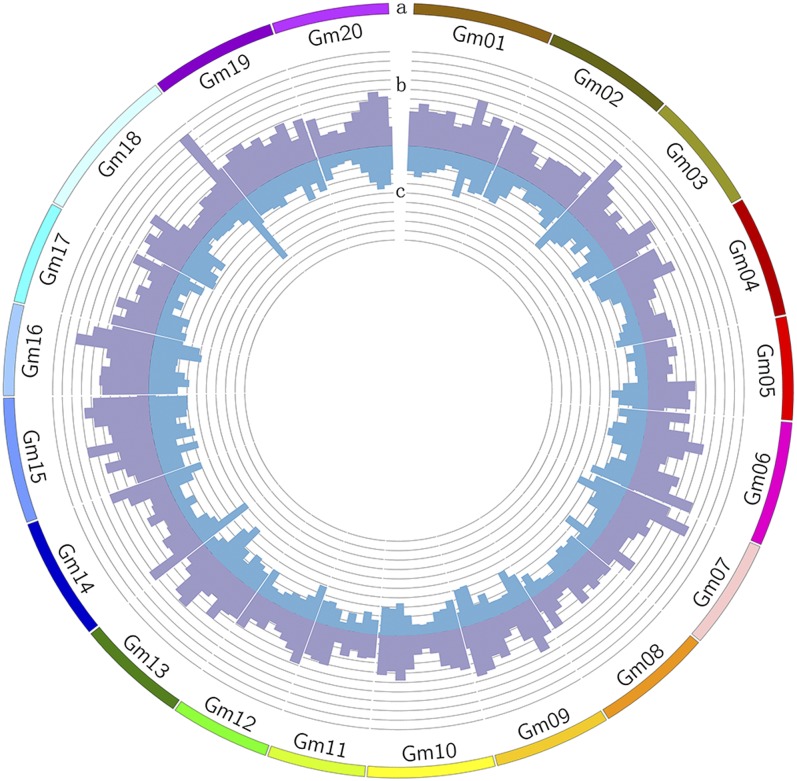
Distribution of INDELs identified between Williams 82 and Hedou 12 along 20 chromosomes. Circular representation of the distribution of insertions and deletions in the soybean genome. (A) The 20 chromosomes of *Glycine max* (Gm), (B) The number of deletions in sliding windows of 5 Mb in each chromosome of *G. max*, (C) The number of insertions in sliding windows of 5 Mb in each chromosome of *G. max*. Scale is 40 INDELs between neighbor concentric circles.

**Figure 2:**
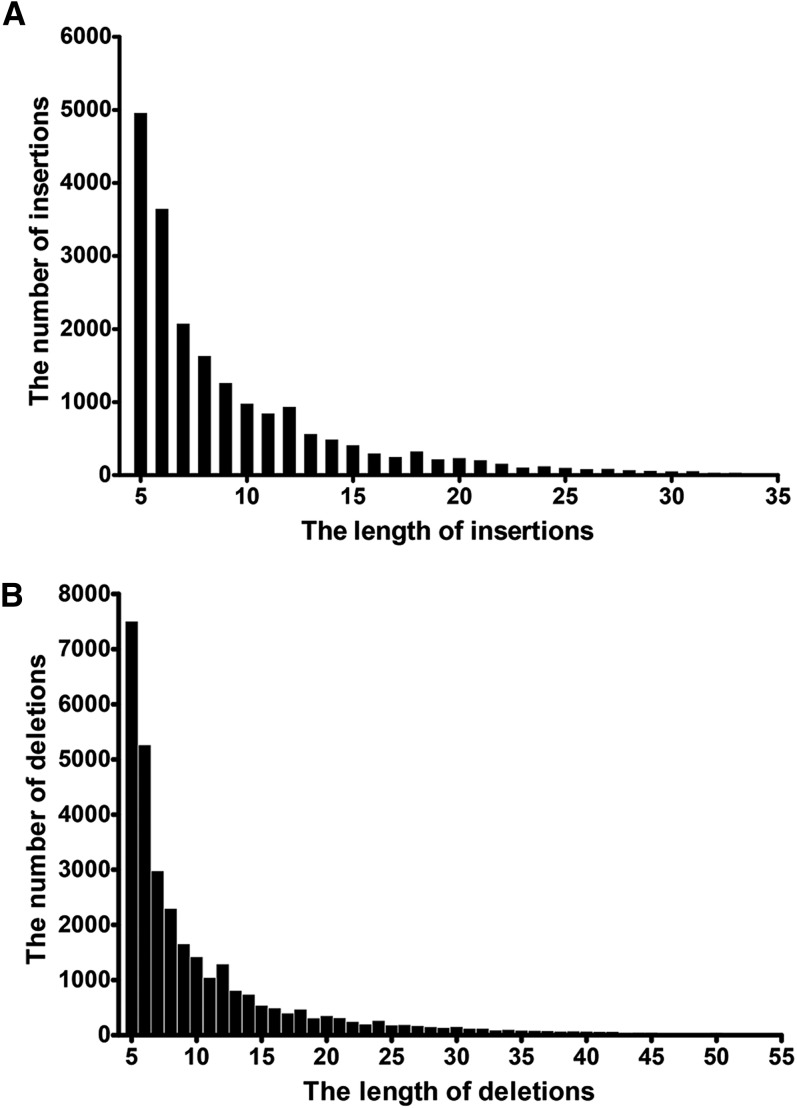
The size distribution of insertions (A) and deletions (B) range from 5 to 55 bp between Williams 82 and Hedou 12. The horizontal axis represents the size of the insertion or deletion. The vertical axis represents the number of insertions or deletions.

The frequency of insertions and deletions across the 20 soybean chromosomes is shown in Figure 1A. The number of detected INDELs on each chromosome ranged from 1466 (Gm11, 34.7 Mb in length) to 3397 (Gm15, 51.7 Mb in length) (Figure 1B). 23,545 of 48,613 INDELs were pericentromeric (information regarding the pericentromeric region was collected from the Soybean Genome Browser at (http://SoyBase.org/gb2/gbrowse/gmax1.01/). The genome-wide average density was about 52.4 INDELs per Mb. Insertions and deletions 5 bp in length occurred most often (4397 and 7477 cases, respectively), whereas longer insertions or deletions were rare (Figure 2).

### Validation of INDEL polymorphisms between Hedou 12 and Williams 82

**Figure 3:**
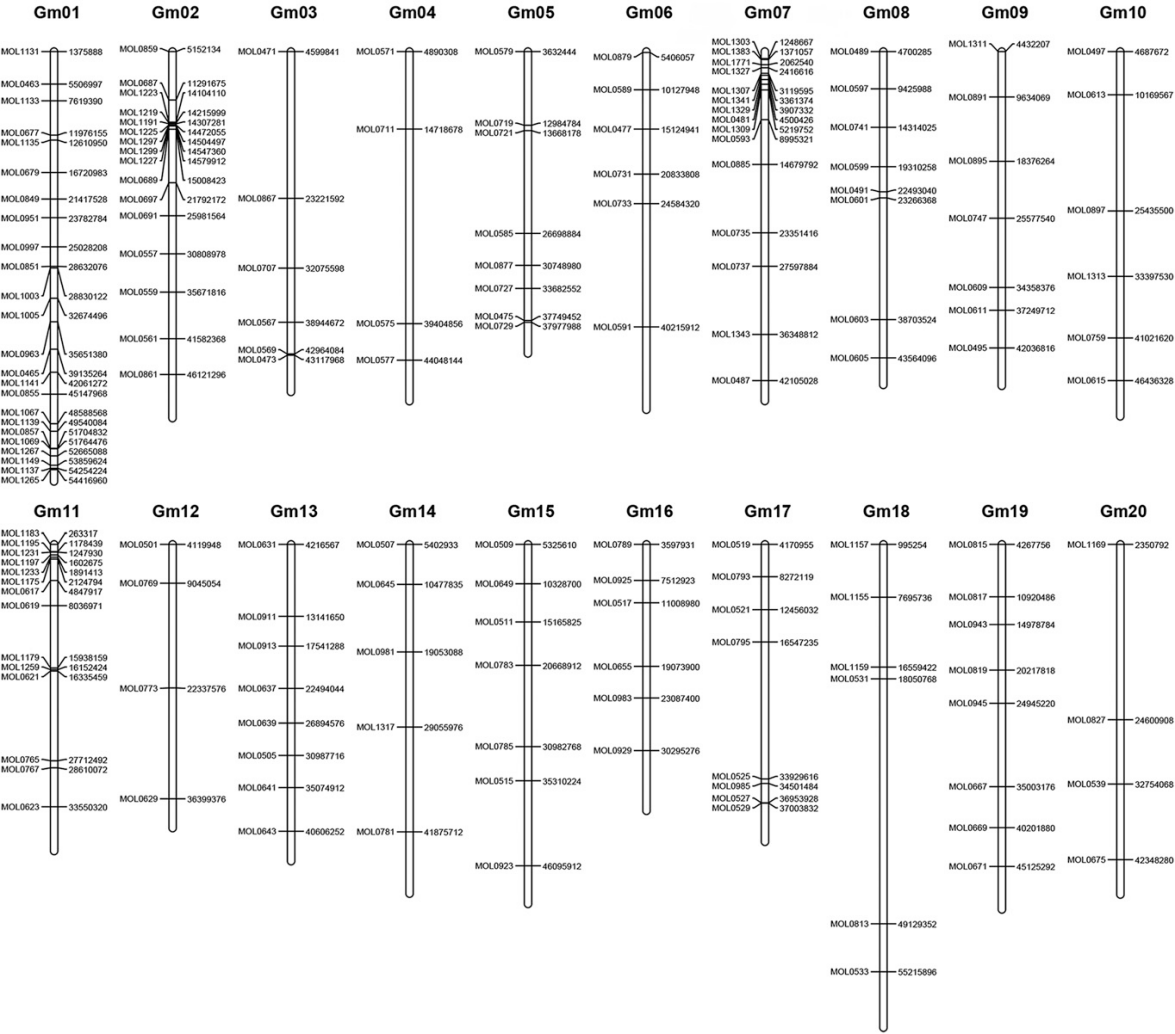
Physical map positions of 170 INsertion/DELetion markers in the soybean genome. Marker names are indicated on the left side and physical positions on the right side of each chromosome.

We performed PCR amplification to confirm the selected INDEL markers between Hedou 12 and Williams 82. Out of 243 primer sets of candidate INDEL markers, 237 produced reliable PCR products in both Hedou 12 and Williams 82, while the remaining six primer sets failed to amplify any PCR products. Of these 237 tested INDEL makers, 165 (68%) showed clear polymorphisms as predicted between Hedou 12 and Williams 82, and the rest produced either PCR products of the same size or unpredicted polymorphisms. The validated 165 INDEL markers were distributed throughout the whole genome (Figure 3). The number of validated INDEL markers ranged from 4–24 per chromosome. These validated INDEL markers included 132 designed anchor markers and 33 markers in a 37.3 Mb region from three chromosomes that was selected for testing the utility of the INDEL markers for fine mapping. In the fine-scale analysis, we obtained 18, 9, and 6 validated INDEL markers on Gm01, Gm02, and Gm11, respectively. We did not find difference in success rates for validations between large and fine-scale marker development. These results indicated that INDELs could be used as both anchor markers and fine mapping markers (information regarding the above INDEL markers, including primer sequences, PCR product size, and physical position, is provided in Supporting Information, Table S1).

### Characteristics of INDEL polymorphisms in soybean cultivars

The 165 validated INDEL markers were also tested in 12 other soybean cultivars, including eight Chinese cultivars: Jilin 35, Wandou 28, Jindou 21, Fendou 33, Shidou 111, Andou 1311, Zhonghuang 30, and Zhoudou 30; and four Americans: Jack, Essex, Forrest and HVE. The polymorphisms in the INDEL markers among the 14 evaluated cultivars are shown in Table S2. Most of the INDEL markers (92%, 151/165) produced two allele products, and the remaining 14 markers detected a third allele in one to four cultivars respectively. 40 markers amplified no products in some cultivars, probably owing to polymorphisms at the primer binding sites in these cultivars compared with the reference genome sequence from which the primers were derived.

**Figure 4:**
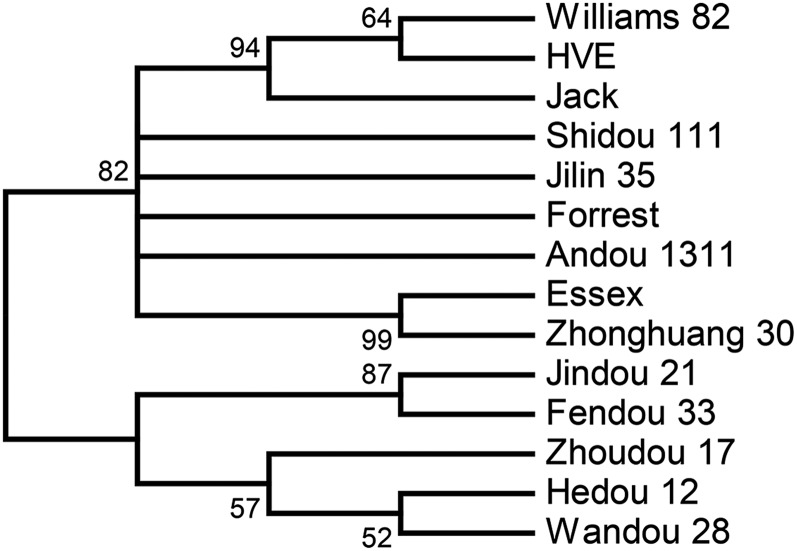
Dendrogram of unweighted pair group method analysis (UPGMA) clustering analysis of 14 soybean cultivars.

Polymorphism rates between these 14 cultivars based on 165 INDEL markers are shown in Table 1. There were more polymorphisms between Hedou 12 and the American cultivars (Williams 82, Jack, HVE, Essex, and Forrest) than between Williams 82 and the Chinese cultivars. The lowest number of polymorphisms was observed between Jindou 21 and Fendou 33, with 27 polymorphic markers. Jilin 35, Andou 1311, Shidou 111, and Zhonghuang 30 were found to be closer to the American cultivars than to the other Chinese cultivars, as indicated by a cluster analysis using MEGA 5.2.1 (Figure 4). The second largest numbers of polymorphisms in the pairwise comparison came from a Chinese cultivar and two American cultivars: Hedou 12 and HVE/Jack with 130 varieties (79% of the total INDEL markers). The results indicated that these INDEL polymorphisms were widely transferable. Cluster analysis of the polymorphisms of these 14 cultivars revealed two groups: one group with Chinese cultivars only and the other group with both Chinese and American cultivars. This result suggests that some Chinese cultivars share common ancestors with American cultivars; the others probably having unique ancestors in Chinese breeding history.

**Table 1:** Number of INsertion/DELeletion markers found to be polymorphic in pairwise comparisons of 14 soybean cultivars

	Hedou 12	Williams 82	Jilin 35	Jack	HVE	Essex	Forrest	Wandou 28	Jindou 21	Fendou 33	Shidou 111	Andou 1311	Zhonghuang 30	Zhoudou 17
**Hedou 12**		165	102	130	130	94	104	66	72	83	82	76	94	71
**Williams 82**	100%		67	31	36	69	63	102	91	75	70	78	70	92
**Jilin 35**	62%	41%		59	61	56	58	80	69	71	67	78	78	67
**Jack**	79%	19%	36%		40	58	56	87	71	67	68	75	66	76
**HVE**	79%	22%	37%	24%		52	64	96	85	81	61	79	76	83
**Essex**	57%	42%	34%	35%	32%		48	65	70	68	62	53	75	68
**Forrest**	63%	38%	35%	34%	39%	29%		72	80	70	73	58	70	64
**Wandou 28**	40%	62%	48%	53%	58%	39%	44%		64	65	71	61	93	46
**Jindou 21**	44%	55%	42%	43%	52%	42%	48%	39%		27	68	66	69	52
**Fendou 33**	50%	45%	43%	41%	49%	41%	42%	39%	16%		61	61	66	57
**Shidou 111**	50%	42%	41%	41%	37%	38%	44%	43%	41%	37%		84	69	64
**Andou 1311**	46%	47%	47%	45%	48%	32%	35%	37%	40%	37%	51%		73	65
**Zhonghuang 30**	57%	42%	47%	40%	46%	45%	42%	56%	42%	40%	42%	44%		78
**Zhoudou 17**	43%	56%	41%	46%	50%	41%	39%	28%	32%	35%	39%	39%	47%	

The percentages of polymorphisms in pairwise comparisons of 14 soybean cultivars are shown below the diagonal.

### Fine mapping of *crinkly leaf* with INDEL markers

**Figure 5:**
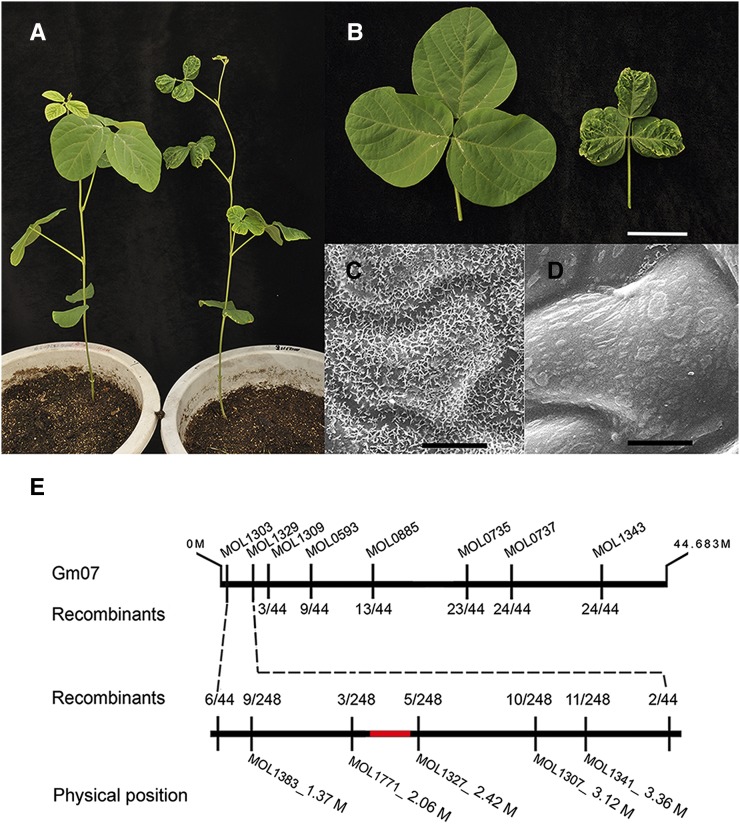
Phenotypes of the *crinkly leaf* mutant and fine-mapping of the mutated locus. (A) Whole plant of the *crinkly leaf* mutant (right), compared with the wild-type Hedou 12 (left) 40 days after sowing. (B) The third compound leaf of wild-type Hedou 12 (left) and the *crinkly leaf* mutant (right) from 40-day-old plants. Scale bar, 4 cm. Scanning electron microscope analysis for mature adaxial leaf epidermal cells of the wild type Hedou 12 (C) and the *crinkly leaf* mutant (D). Scale bar in (C) and (D), 8.6 μm. (E) Mapping of the *crinkly leaf* mutant. The upper horizontal line represents soybean chromosome Gm07, INsertion/DELetion markers and the number of recombinants between the marker and *CRINKLY LEAF* locus are shown above and below vertical lines. The red horizontal line represents the deletion region.

To evaluate the usability of the above developed markers for genetic mapping, we chose a *crinkly leaf* mutant from the Hedou12 mutagenesis population. In the *crinkly leaf* mutant, the tip of each leaflet became necrotic during the early phase of leaf development while the other parts of the leaflet continued to grow. This produced the mutant’s distinctive crinkly leaves (Figure 5, A and B). The wild-type Hedou 12 leaflet had three times the area and twice the dry weight compared with the *crinkly leaf* leaflet. Mature adaxial leaf epidermal cells of *crinkly leaf* and Hedou 12 were analyzed by scanning electron microscopy (Figure 5, C and D). The mutant had an abnormal crystal wax layer deposited on the surface of the epidermal cells.

The *crinkly leaf* mutant was crossed with Williams 82 to produce a five-plant F1 population and led to an F2 mapping population of 511 plants. Of these, 124 plants showed mutant phenotypes in the F2 generation with a frequency of ∼0.25. This was consistent with a single recessive mutation with a chi-square value of χ^2^ =0.147 (P value > 0.5). For preliminary mapping, 22 mutant plants were used to perform genotyping with the above validated 165 INDEL markers. Two INDEL markers on chromosome 7, MOL1303 (six recombination events), and MOL1329 (two recombination events) were found to be closely linked to the mutant phenotype, and the initial 2.67 Mb interval was delimited. Five more markers, MOL1383, MOL1771, MOL1327, MOL1307, and MOL1341 between MOL1303 and MOL1329, were employed for fine-scale mapping in all 124 mutant plants. Then the mutated region was narrowed down to 360 kb between markers MOL1771 and MOL1327 as shown in Figure 5E.

While further internal INDEL markers near MOL1327 were developed, we failed to amplify any specific PCR product. This indicated that there was probably a deletion in this region. We then performed an inverse PCR to investigate the border of the deletion region (Figure S1). Sequencing of the resulting PCR product revealed that there is a 253 kb deletion in the Gm07 from 2,118,557 bp to 2,371,744 bp (Figure 5E, Figure S1). Within this region, 29 genes were predicted by SoyBase, involved in fatty acid metabolism, plasma membrane H^+^ transportation, plant hormone mediated signaling pathways, and auxin polar transport.

In this study, we used INDEL markers to perform fine mapping based on whole genome sequence information. With this method, only a small number of validated anchor markers are required for preliminary mapping, and the markers for fine mapping can be easily developed from detected polymorphisms from NGS in the target region. This is more economical for marker development compared with traditional genetic mapping studies that require the development of a large number of molecular markers to construct a genetic map for further mapping.

## Supplementary Material

Supporting Information
