# Supplementary material for: Development of INDEL Markers for Genetic Mapping Based on Whole Genome Resequencing in Soybean
Source: G3 (Bethesda). 2015 Oct 23;5(12):2793–9. doi: 10.1534/g3.115.022780 (PMC4683650; doi:10.1534/g3.115.022780)
Supplement: Supporting Information [file supp_5_12_2793__index.html]

Development of INDEL Markers for Genetic Mapping Based on Whole-Genome Re-sequencing in Soybean — Development of INDEL Markers for Genetic Mapping Based on Whole Genome Resequencing in Soybean — Supporting Information 

# Development of INDEL Markers for Genetic Mapping Based on Whole Genome Resequencing in Soybean

## Supporting Information for Song *et al.*, 2015

**Files in this Data Supplement:**

- Figure S1 - Determination of deletion border of the *crinkly leaf* mutant. (.doc, 1,232 KB)
- Table S1 - Information of the validated INDEL markers. (.xls, 51 KB)
- Table S2 - Allele polymorphisms in 165 INDEL markers among 14 soybean cultivars. (.xls, 38 KB)
