# Supplementary material for: Development of INDEL Markers for Genetic Mapping Based on Whole Genome Resequencing in Soybean
Source: G3 (Bethesda). 2015 Oct 23;5(12):2793–9. doi: 10.1534/g3.115.022780 (PMC4683650; doi:10.1534/g3.115.022780)

**Figure S1**. Determination of deletion border of the *crinkly leaf* mutant.

(A) Electrophoretograms of PCR products for inverse PCR by a pair of primers OL2578 and OL2579. WT represents Hedou12 wild type, Mutant represents *crinkly leaf* mutant. M represents DL2000 DNA marker (Takara).

(B) Electrophoretograms of PCR products for validation of the inverse PCR by a pair of primers OL2623 and OL2624. WT represents Hedou12 wild type, Mutant represents *crinkly leaf* mutant. M represents DL2000 DNA marker (Takara).

(C) The sequence of the deletion validation PCR products of *crinkly leaf* mutant.

The position of the 253Kb deletion was indicated in red box.

(D) The diagrammatic sketch of the 253Kb deletion in *crinkly leaf* mutant in the genome region of chromosome 7 from Soybase. The red box shows the deletion region.


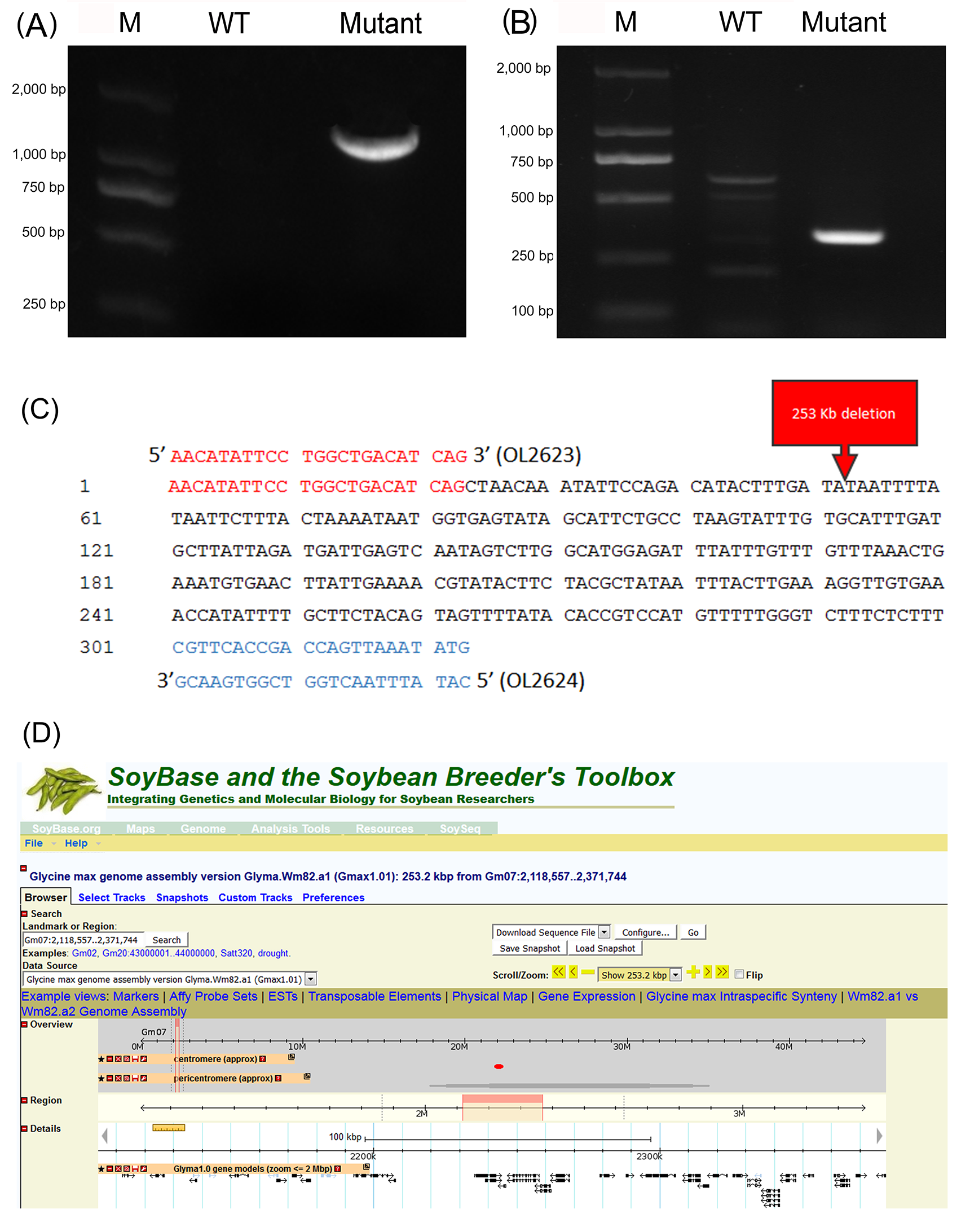

Supplement: Supporting Information [file supp_g3.115.022780_FigureS1.doc]
